# Expression Dynamics and Protein Localization of Rhabdomeric Opsins in *Platynereis* Larvae

**DOI:** 10.1093/icb/ict046

**Published:** 2013-05-10

**Authors:** Nadine Randel, Luis A. Bezares-Calderón, Martin Gühmann, Réza Shahidi, Gáspár Jékely

**Affiliations:** Max Planck Institute for Developmental Biology, Spemannstrasse 35, 72076 Tuebingen, Germany

## Abstract

The larval stages of polychaete annelids are often responsive to light and can possess one to six eyes. The early trochophore larvae of the errant annelid *Platynereis dumerilii* have a single pair of ventral eyespots, whereas older nectochaete larvae have an additional two pairs of dorsal eyes that will develop into the adult eyes. Early *Platynereis* trochophores show robust positive phototaxis starting on the first day of development. Even though the mechanism of phototaxis in *Platynereis* early trochophore larvae is well understood, no photopigment (opsin) expression has yet been described in this stage. In late trochophore larvae, a rhabdomeric-type opsin, *r-opsin1*, expressed in both the eyespots and the adult eyes has already been reported. Here, we identify another *Platynereis* rhabdomeric opsin, *r-opsin3*, that is expressed in a single photoreceptor in the eyespots in early trochophores, suggesting that it mediates early larval phototaxis. We also show that *r-opsin1* and *r-opsin3* are expressed in adjacent photoreceptor cells in the eyespots in later stages, indicating that a second eyespot-photoreceptor differentiates in late trochophore larvae. Using serial transmission electron microscopy (TEM), we identified and reconstructed both photoreceptors and a pigment cell in the late larval eyespot. We also characterized opsin expression in the adult eyes and found that the two opsins co-express there in several photoreceptor cells. Using antibodies recognizing r-opsin1 and r-opsin3 proteins, we demonstrate that both opsins localize to the rhabdomere in all six eyes. In addition, we found that *r-opsin1* mRNA is localized to, and translated in, the projections of the adult eyes. The specific changes we describe in opsin transcription and translation and in the cellular complement suggest that the six larval eyes undergo spectral and functional maturation during the early planktonic phase of the *Platynereis* life cycle.

## Introduction

Positive phototaxis of early larval stages is a widespread phenomenon among marine invertebrates, characteristic of approximately 80% of the benthic invertebrates with a pelagic larva (Thorson 1964). Simple larval eyes, sometimes consisting of only a single photoreceptor cell with shading pigment (Nordström et al. 2003), or a photoreceptor cell and a pigment cell (Jékely et al. 2008), are known to mediate larval phototaxis. Simple larval eyes are widespread among marine invertebrates and have been characterized morphologically in the larval stages of sponges (Leys and Degnan 2001), cnidarians (Nordström et al. 2003), annelids (Bartolomaeus 1992a), mollusks (Bartolomaeus 1992b), nemerteans (Döhren and Bartolomaeus 2007), flatworms (Eakin and Brandenburger 1981), brachiopods (Passamaneck et al. 2011), hemichordates (Brandenburger et al. 1973), cephalochordates (Lacalli 1996), and crustaceans (Lacalli 2009). In contrast to the wealth of morphological studies, few studies have focused on the molecular and functional characterization of larval eyes (Jékely et al. 2008; Passamaneck et al. 2011; Vopalensky et al. 2012). A better understanding of the molecular and functional characteristics of the eyes of larval marine invertebrates would allow a more reliable reconstruction of early stages of evolution of eyes in bilaterians, because the eyes of some larvae may have retained an ancestral organization (Arendt and Wittbrodt 2001; Jékely et al. 2008). In particular, opsins, the seven-transmembrane G-protein-coupled receptors responsible for light detection, have received most attention in comparative studies of photoreceptors. Opsins are phylogenetically stable markers of photoreceptor types and their molecular divergences may have paralleled the functional divergence of photoreceptors (Arendt 2003). Divergences of opsins also contribute to the evolution of spectral diversity among photoreceptors (Porter et al. 2009).

The larval eyespots of the marine annelid *Platynereis dumerilii* are among the most well-characterized simple larval eyes (Jékely et al. 2008). *Platynereis* eyespots develop laterally in the episphere of the early trochophore larva (for staging see Fischer et al. 2010) and are present throughout larval stages, and might persist in the adult as frontolateral eyelets (Backfisch et al. 2013). The eyespots consist of a rhabdomeric photoreceptor cell and an associated shading pigment cell (Rhode 1992), and mediate positive phototaxis of the helical-swimming larva from 24 hours post fertilization (hpf) onward (developing at 18 °C). The axon of the eyespot photoreceptor directly synapses on the ipsilateral multi-ciliated cells of the ciliary band (prototroch) and upon stimulation, cholinergic signals from the photoreceptor reduce the frequency of ciliary beats and change the ciliary stroke pattern in the adjacent prototroch cells (Jékely et al. 2008). These changes are triggered at every 180° rotation by the alternating illumination of the two eyespots and steer the helical trajectories toward the light source. Despite these advances, no opsin has yet been described in the eyespots of the early *Platynereis* trochophore.

In late trochophore larvae (40–48 hpf), two additional pairs of eyes start to develop in the dorsal episphere (Rhode 1992; Arendt et al. 2002). The newly developing eyes represent the precursors of the adults’ pigment-cup eyes (henceforth referred to as ‘adult eyes’, also when discussing larval stages). These are everse, cerebral eyes consisting of several pigment and photoreceptor cells, the number of which increases with age. The adult eyes also have a lens, formed by the apical protrusions of the pigment cells. The photoreceptor rhabdomeres are located inside the pigment cup (Rhode 1992). The adult eyes express a rhabdomeric opsin (r-opsin), *r-opsin1* (Arendt et al. 2002). This opsin is also expressed in the eyespots after 2 days post fertilization (dpf), long after the onset of phototactic behavior (Arendt et al. 2002). Three other *Platynereis* opsins *r-opsin2* (D. Arendt, EMBL, personal communication), *r-opsin3*, and *r-opsin-4* have been identified, but their expression patterns have not yet been characterized.

Here, we characterize the expression of *Platynereis r-opsin3* and characterize its co-expression with *r-opsin1* both at the transcript level and at the protein level. Using serial TEM, we also reconstruct the morphology of the cellular complements of the eyespots in the nectochaete larva. Our results indicate a spectral and functional maturation of the eyes of the *Platynereis* nectochaete larva.

## Materials and methods

### Molecular phylogeny

We selected a subset of arthropod and lophotrochozan r-opsin sequences from the NCBI non-redundant and EST databases, and from the *Capitella teleta* and *Lottia gigantea* genome projects at JGI (http://genome.jgi.doe.gov/). r-Opsin protein sequences were aligned with MUSCLE (Edgar 2004). The profile thus obtained was merged to an alignment of ciliary opsins using PRANK (Loytynoja and Goldman 2008). The resulting alignment was cropped at both ends to remove poorly aligned regions.

We inferred opsin phylogeny using two methods. First, we performed a maximum-likelihood analysis using PhyML3.0 (Guindon et al. 2010) and the aLRT statistics to evaluate the likelihood of each node. We used an LG + G + I model and the best of NNI + SPR search strategies for the inference. Next, we ran a Bayesian analysis with MrBayes 3.2 (Ronquist et al. 2012) using the following parameters: two million generations with sampling each 100 generations, four chains per each of two independent runs, a chain temperature of 0.25, and an initial burn-in fraction of 0.5. We estimated the substitution model, the gamma parameter, and invariant sites. We stopped the analysis after the average standard deviation of split frequencies was equal to or less than 0.01. The resulting analysis reached this point after 220 000 generations. After analyzing convergence criteria with Tracer and with the compare function of AWTY (Nylander et al. 2008), we summarized the parameters and trees with a burn-in fraction of 0.5 (11 100 samples).

### Generation of antibodies

Rabbits were immunized with synthetic peptides (CSEVLVPGSMSLDGLLTTAH for r-opsin1 and CSKPQAAPKGKGGKDN for r-opsin3) coupled to an adjuvant (lipoadjuvant Pam3) via an N-terminal Cys. Sera were affinity-purified on the respective peptide epitopes coupled to a SulfoLink resin (Thermo Scientific, Rockford, IL), as described by Conzelmann and Jékely (2012). Upon elution, we fractionated the bound antibodies using a 100 mM glycine buffer of decreasing pH (4.7 to 2.0). For the r-opsin3 antibody, the yield of high-affinity antibody was very low; therefore, we are unable to provide it.

### Staining of tissues

Single and double *in situ* hybridization and immunostaining were performed as previously described (Tessmar-Raible et al. 2005; Conzelmann and Jékely 2012).

### Transmission electron microscopy

Seventy-two-hpf *Platynereis* larvae were fixed using a high-pressure freezer (BAL-TEC HPM 010, Balzers, Liechtenstein) and transferred to liquid nitrogen. Frozen samples were cryosubstituted with 2% osmium tetroxide in acetone and 0.5% uranyl acetate in a cryosubstitution unit (Leica EM AFS-2) over a regime of gradually rising temperatures. Samples were embedded in Epon. Fifty nanometer (nm) serial sections were cut on a Reichert Jung Ultracut E microtome. The sections were collected on single-slotted copper grids (NOTSCH-NUM 2 × 1 mm, Science Service, München) with Formvar support film, contrasted with uranyl acetate and Reynold’s lead citrate, and carbon coated to stabilized the film. Image acquisition of serial sections was performed at a pixel size of 3.72 nm on a FEI TECNAI Spirit transmission electron microscope equipped with an UltraScan 4000 4X4k digital camera using the image acquisition software Digital Micrograph (Gatan Software Team Inc., Pleasanton). Stitching and alignment were accomplished using TrakEM2 (Cardona et al. 2010). All structures were segmented manually as area-lists, and exported into 3Dviewer and Blender.

## Results

### Identification of a novel r-opsin in *P. dumerilii*

In an unpublished mixed-stages *Platynereis* EST library (available at http://jekely-lab.tuebingen.mpg.de/), we identified a partial and a full-length clone encoding two different rhabdomeric-type opsins. Using Rapid Amplification of cDNA Ends (RACE), we obtained the missing coding region and the 5′-UTR for the partial clone, allowing us to isolate a full-length sequence from *Platynereis* cDNA. Both cDNAs encode an opsin protein with seven predicted transmembrane helices and a conserved lysine in the seventh transmembrane helix, a defining feature of opsins (Terakita 2005). Following the seventh transmembrane helix, the opsins have a highly conserved histidine–proline–arginine (HPR) tripeptide motif that is shared with other r-opsins (HPK or HPR) (Arendt 2004), identifying them as r-opsins. We named these *Platynereis* opsin genes *r-opsin3* (KC810971) and *r-opsin4* (KC810972). We will refer to the first identified r-opsin as *r-opsin1* (Arendt et al. 2002).

**Fig. 1:**
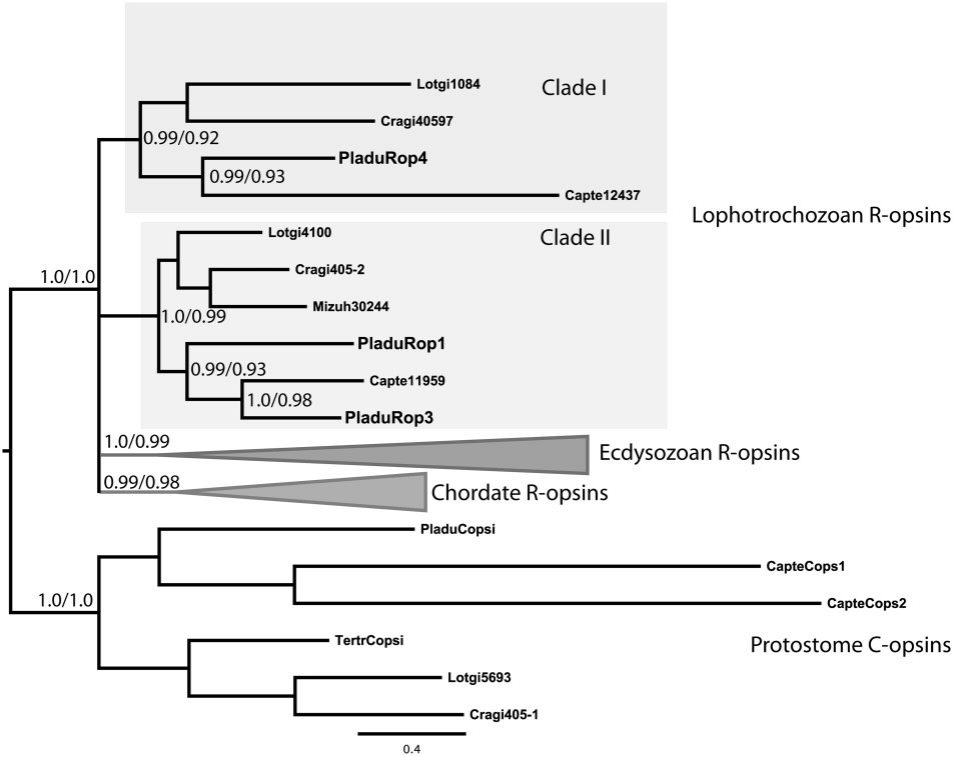
Phylogenetic tree of protostome r-opsins. Bayesian phylogenetic tree inferred from a protein alignment of protostome r-opsins and c-opsins. The tree shows that r-opsin1 and r-opsin3 diverged early during the evolution of annelids. Posterior probabilities of a Bayesian phylogeny (Bayesian posterior probability/aLRT value) and bootstrap values of a maximum-likelihood tree are indicated at the main nodes.

To better understand the relationship of r-opsin3 and r-opsin4 to other r-opsins, we performed molecular phylogenetic analyses. In a tree of select opsins, r-opsin3 clustered together with high support with an opsin from the sedentary annelid *C. teleta*. The r-opsin1 sequence was an outgroup to the r-opsin3 sequences (Fig. 1). This tree topology indicates that the duplication event giving rise to the *r-opsin1* and *r-opsin3* paralogs predates the divergence of the two major annelid lineages, Errantia and Sedentaria (Struck et al. 2011). These opsins formed a well-supported clade (Clade II) with some mollusk r-opsins. We could identify another stable r-opsin clade (Clade I) with both mollusk and annelid sequences. This clade includes the *Platynereis r-opsin4* and a *C. teleta* r-opsin.

### Spatial and temporal pattern of expression of *r-opsin3* and *r-opsin1* in *Platynereis* larval stages

**Fig. 2:**
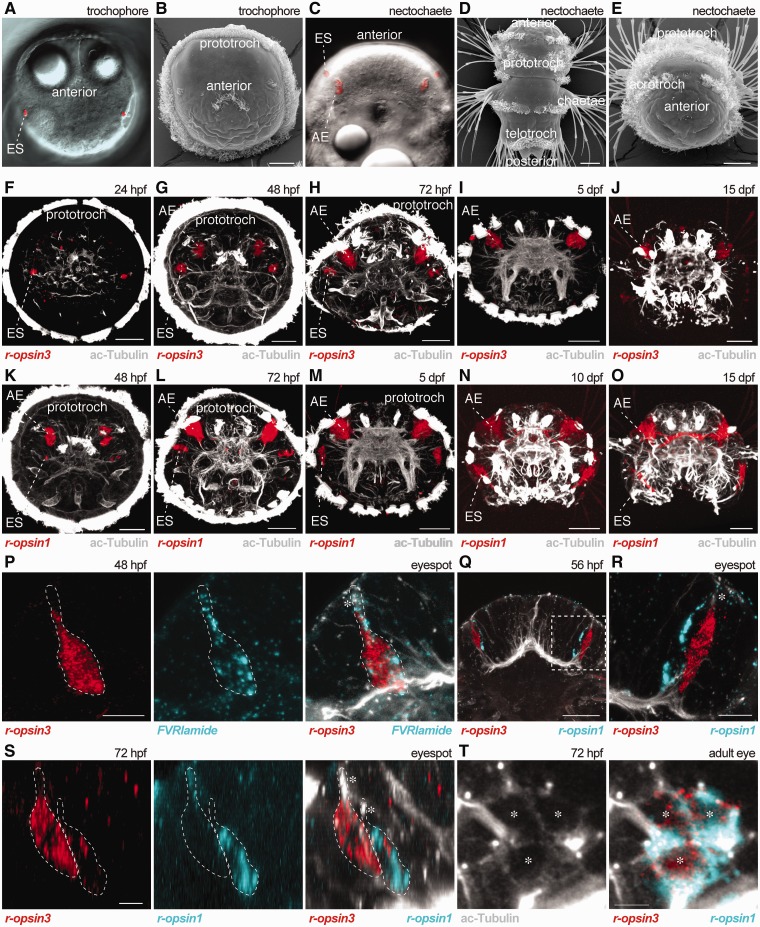
Expression of *r-opsin3* and *r-opsin1* in *Platynereis* trochophore and nectochaete larvae. (**A**) Differential interference contrast (DIC) image of a 48-hpf larva with eyespots (red). (**B**) SEM image of a trochophore (48 hpf) larva. (**C**) DIC image of a 72-hpf larva with eyespots and adult eyes (red). (**D**, **E**) SEM images of nectochaete larvae (72 hpf) oriented dorsally (D) or anteriorly (E). (**F–J**) *In situ* hybridization in the indicated larval stages for *r-opsin3* (red) counterstained for acetylated tubulin (white). (**K–O**) *In situ* hybridization in the indicated larval stages for *r-opsin1* (red) counterstained for acetylated tubulin (white). (**P**) Double *in situ* hybridization for *r-opsin3* (red) and *FVRIamide precursor* (cyan) counterstained for acetylated tubulin (white) in a 48-hpf larva reveals co-expression in the photoreceptor cell of the eyespot. (**Q–T**) Double *in situ* hybridization for *r-opsin3* (red) and *r-opsin1* (cyan) counterstained for acetylated tubulin (white) in 56-hpf (Q, R) and 72-hpf (S, T) larvae reveals the expression of the two opsins in two adjacent photoreceptor cells of the eyespot (Q–S), and co-expression in the adult eye (T). In (A, C), the eyespot’s pigment was highlighted using the reflection of the laser light in the confocal microscope. In (P–S), asterisks label the dendrites of the photoreceptor cells, in (T) the cell bodies of the photoreceptors. In (P, S), dashed lines mark the boundaries of the photoreceptor cells as revealed by the *in situ* and the acetylated-tubulin signals. The boxed area in (Q) is shown enlarged in (R). AE, adult eye; ES, eyespot. Scale bars: (B, D, E–O, Q): 30 µm, (P, R, S, T) 5 µm.

To analyze the expression of *r-opsin3* during the development of *Platynereis*, we performed whole-mount *in situ* hybridization on various larval stages and adults (Fig. 2A–E). In contrast to *r-opsin1*, which is not expressed until the onset of differentiation of the photoreceptor in the adult eye at 43 hpf (Arendt et al. 2002), we detected prominent expression of *r-opsin3* already in 24-hpf larvae (Fig. 2F). The *r-opsin3* is expressed in single cells in the position of the eyespots. The *in situ* signal in each cell is associated with an apical dendrite labeled by acetylated-tubulin.

To characterize the expression of *r-opsin3* in the eyespots at cellular resolution, we performed double *in situ* hybridization with *r-opsin3-*specific and *FVRIamide-precursor*-specific probes. *FVRIamide precursor* is a proneuropeptide gene expressed in the eyespot photoreceptor in early *Platynereis* larvae (Jékely et al. 2008). An antibody against the active FVRIamide neuropeptide labels the axonal projection of the photoreceptor cell to the ciliary band (Jékely et al. 2008). Confocal microscopic imaging of double *in situ* hybridization samples revealed cellular co-expression of the two genes in 24-hpf larvae (Fig. 2P). We also detected the expression of *r-opsin3* in the eyespots in 48-hpf and 72-hpf larvae, but not in 5-dpf and 15-dpf larvae (Fig. 2G–J). The *r-opsin3* is also expressed in the adult eyes from 48 hpf onward including adult stages (Fig. 2G–J, Supplementary Fig. S1). We could also detect expression of *r-opsin3* in the adult notopodia, in a position similar to that recently described for *r-opsin1* (Supplementary Fig. S1) (Backfisch et al. 2013).

**Fig. 3:**
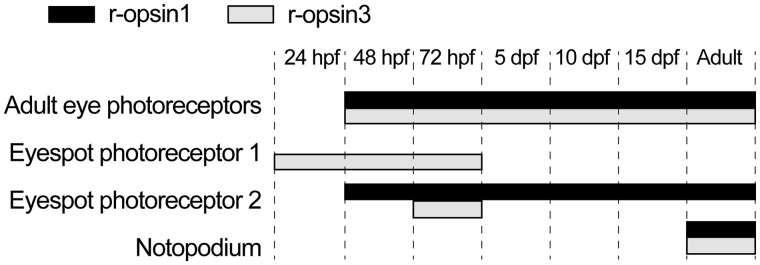
Summary diagram of the expression patterns of *Platynereis r-opsin1* and *r-opsin3* in the photoreceptor cells of the adult eyes, eyespots, and parapodia in various larval and adult stages, based on the data in this article and Backfisch et al. (2013).

In contrast to *r-opsin3*, *r-opsin1* is not expressed in the eyespots until the late trochophore stage (Arendt et al. 2002) (Fig. 2K). We also found strong *r-opsin1* expression in the eyespots at 15 dpf indicating that *r-opsin1* expression is maintained longer than that of *r-opsin3* (Fig. 2L–O). Expression of *r-opsin1* is strong in the adult eyes from 48 hpf onward and could also be detected in juveniles and adults (Figs. 2K–O and 3).

The late onset of *r-opsin1* expression in the eyespots suggested that the two opsins could co-express in the eyespot photoreceptor. To test this, we performed double *in situ* hybridization and confocal microscopy imaging experiments with *r-opsin1* and *r-opsin3*. We found that the two opsins are expressed in two adjacent cells in the eyespots in 56-hpf late trochophore larvae with *r-opsin1* labeling the more medial cell (Fig. 2Q and R). In 72-hpf nectochaete larvae, weak *r-opsin3* expression could also be detected in the more medial cell, co-expressing with *r-opsin1* (Fig. 2S). These findings were surprising, since previous ultrastructural work revealed the presence of only one photoreceptor in the eyespot. In contrast to the eyespots, in the adult eyes, we detected co-expression of the two opsins in the same photoreceptor cells (Fig. 2T). Co-expression of the two opsins in single photoreceptor cells was verified by detecting the cell borders with acetylated tubulin staining. The two signals from the double *in situ* hybridization show little overlap within the same cell due to the strong shadowing effect of the NBT/BCIP precipitate (Jékely and Arendt 2007). We could not detect the expression of r-opsin4 in various larval stages using *in situ* hybridization.

### Serial TEM reconstruction of the morphology of the eyespot in a nectochaete larva

**Fig. 4:**
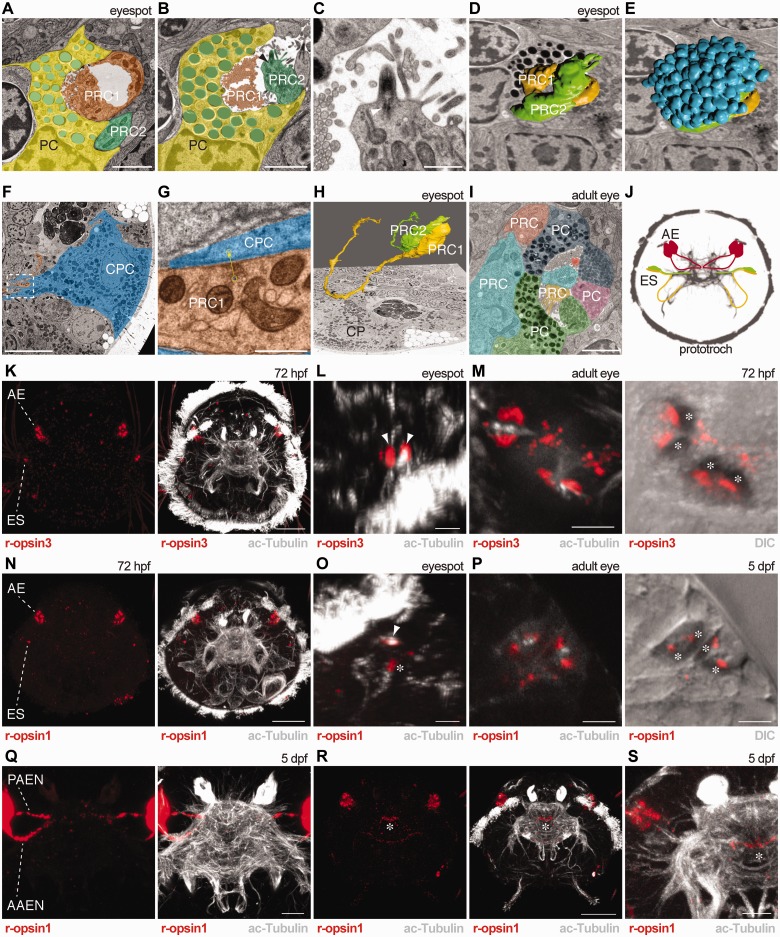
Ultrastructure of eyes and immunohistochemical localization of opsins in *Platynereis* rhabdomeric photoreceptors. (**A–H**) Ultrastructure of an eyespot and the projection of the photoreceptor cells in a 72-hpf larva. (A, B) TEM images of an eyespot with the two rhabdomeric photoreceptor cells (orange, green), the pigment cell (yellow) and pigment granules (cyan) in two different layers. (C) TEM image of the apical end of a photoreceptor cell with a cilium. (D, E) TEM images of the eyespot with the reconstructed 3D structure of the two rhabdomers (D) and the pigment granules (E). (F, G) TEM images of the prototroch region. (F) Axon of a photoreceptor cell (orange) in close contact with a prototroch cell (cyan). (G) Close-up image of a synapse between the photoreceptor cell (orange) and the prototroch cell (cyan). (H) TEM image of the prototroch region with the reconstructed photoreceptor cells and their axons. (I) TEM image of an adult eye. Each cell is labeled with a different color. (J) Schematic drawing of the eyes and their projections to the apical nerve plexus. (K–M) Immunostaining for r-opsin3 (red) in 72-hpf larvae counterstained for acetylated tubulin (white). (L) Close-up image of the eyespot, (M) close-up image of the adult eye. In (M), the fluorescent signal is overlaid with the DIC signal in the right panel to show the eye pigment. (N–P) Immunostaining for r-opsin1 (red) in 72-hpf and 5-dpf larvae counterstained for acetylated tubulin (white). (O) Close-up image of the eyespot, (P) close-up image of the adult eye. In (P), the fluorescent signal is overlaid with the DIC signal in the right panel to show the eye pigment. (Q) *In situ* hybridization for *r-opsin1* (red) in a 5-dpf larva counterstained for acetylated tubulin (white) reveals the mRNA signal in the photoreceptor projections in the adult eye. (R) Immunostaining for r-opsin1 (red) in a 5-dpf larva counterstained for acetylated tubulin (white). (S) Close-up image of the right adult eye and the apical nerve plexus. In (B, C), arrowheads indicate the basal body of PRC2 and in (L, O), arrowheads indicate the photoreceptor’s rhabdomeres. Asterisks show the pigment cups (in M, P), the immunostaining signal in the photoreceptor cell-body deeper in the tissue (in O), and the r-opsin1 signal in the apical nerve plexus in the area of the photoreceptor projections (in R, S). AAEN, anterior adult-eye nerve; AE, adult eye; CPC, ciliated prototroch cell; ES, eyespot; PAEN, posterior adult-eye nerve; PC, pigment cell; PRC, photoreceptor cell. Scale bars: (A, B, I) 2 µm, (C, G) 0.5 µm, (F, Q, S) 10 µm, (M) 15 µm, (K, N, R) 30 µm, (L, O, P) 5 µm.

The results of the double *in situ* hybridization indicated that the eyespots differentiate a second photoreceptor in the late trochophore stage. An alternative possibility is that the pigment cell starts to express *r-opsin1.* To distinguish between these two possibilities, we performed serial-sectioning TEM (serial TEM) on 72-hpf nectochaete larvae and examined the ultrastructure of their eyespots. In contrast to the early trochophore larval stage, in which only one photoreceptor had been described in the eyespots (Rhode 1992; Jékely et al. 2008), we identified two photoreceptors and a pigment cell (Fig. 4A–E and H). The distal end of both photoreceptors had parallel microvilli protruding into the cup-shaped depression of the pigment cell (Fig. 4A and B). The photoreceptor with the more laterally located cell body had one cilium; the photoreceptor with the more medial cell body had two cilia with 9 + 0 microtubule doublets and associated basal bodies (Fig. 4C).

It has been shown that the single eyespot photoreceptor cell, expressing *FVRIamide-precursor,* synapses on the prototroch ciliary band in young trochophore larvae (Jékely et al. 2008). To test which of the two photoreceptors identified in the TEM sections corresponds to this cell (i.e., the cell that also expresses *r-opsin3*, based on the results of our double *in situ* hybridization) (Fig. 2P), we traced the axonal projection of both photoreceptors in several serial sections. The lateral cell projected an axon to, and synapses on, the cells of the ciliary band (Fig. 4F–H), identifying it as the *r-opsin3-*expressing photoreceptor. This axon then turns and projects toward the anterior axonal scaffold along the ventral branch of the circumaesophageal connectives (Fig. 4H and J). The axon of the medial, *r-opsin1-*expressing photoreceptor projects along the dorsal branch of the circumaesophageal connectives toward the anterior axonal scaffold (Fig. 4H and J). These results reveal an elaboration of the eyespot circuitry and point at possible functional changes.

### Localization of the opsin proteins to the photoreceptor rhabdomere

Both the adult eye and the eyespot photoreceptors are of the rhabdomeric type and have apical membrane extensions characteristic of these photoreceptors (Rhode 1992). The r-opsins are most commonly localized to apical microvillar extensions in rhabdomeric photoreceptors, with some exceptions such as the vertebrate melanopsins (Hattar et al. 2002) and a sea urchin r-opsin (Ullrich-Lüter et al. 2011), which localize to the photoreceptor cell body, dendrites, and axons or to intracellular vesicles, respectively. Given these exceptions, it was important to test whether both r-opsin1 and r-opsin3 proteins localize to the apical microvilli in the eyes of *Platynereis* larvae. We developed affinity-purified specific antibodies against both r-opsins. Immunolabelings with both antibodies revealed strong and specific signals in the photoreceptor rhabdoms both in the adult eyes and in the eyespots, indicating that the antibodies are specific to the opsins (Fig. 4K–P).

In the eyespots, we could detect r-opsin3 in two structures associated with acetylated-tubulin signal, corresponding to the rhabdomeres of the two photoreceptors, consistent with the results from *in situ* hybridization (Fig. 4K and L). The r-opsin1 could only be detected in one apical structure associated with acetylated-tubulin signal, and also in the body of the photoreceptor cell (Fig. 4N and O).

In the adult eyes, we could detect both opsins in the photoreceptor’s rhabdomeres and cell bodies (Fig. 4K, M, N, and P). Given that the pigment content of the adult eyes was preserved during fixation, we could correlate the antibody signal with the position of the pigment cup (Fig. 4M and P). In agreement with the morphology of the adult eyes (Rhode 1992) (Fig. 4I), the antibodies showed labeling inside the pigment cup, confirming that both opsins are also localized in the photoreceptor’s rhabdomeres (Fig. 4M and P).

Using the r-opsin1 antibody, we also detected protein signal in the median brain of larvae. The signal localized to the area where the photoreceptors of the adult eyes project (Fig. 4R and S) (Backfisch et al. 2013). The photoreceptor projections were revealed by *in situ* hybridizations using the *r-opsin1* probe, also indicating that the *r-opsin1* mRNA is localized to the photoreceptor’s axons (Fig. 4Q), as previously observed (Jékely and Arendt 2007). These results suggest that the *r-opsin1* mRNA is locally translated in the axon terminals of the photoreceptors of the adult eyes.

## Discussion

The identification of a second r-opsin in *Platynereis* broadens our molecular perspective on the light responses by the larvae of this model annelid. The onset of expression of *r-opsin3* in the eyespots correlates with the appearance of positive phototaxis in the larvae. This, together with the localization of the r-opsin3 protein to the photoreceptor’s rhabdomere suggests that r-opsin3 is a functional phototaxis photoreceptor in early trochophore larvae. Given the relatively broad action spectrum (365–545 nm) of phototaxis in early larvae (Jékely et al. 2008), r-opsin3 may not be the only opsin mediating this behavior. The presence of two distinct photoreceptors expressing distinct opsins in the eyespots in later larval stages suggests that in older larvae, the eyespots may develop another function in addition to the regulation of phototaxis. The phototactic function of the eyespot may be maintained until *r-opsin3* is expressed in the ventral eyes. The sustained expression of *r-opsin1* in the eyespot throughout larval and juvenile stages indicates that the eyespots also function in later stages of development. The newly developing *r-opsin1*-expressing photoreceptor of the eyespot projects its axon to the apical nerve plexus rather than to the prototroch cell, suggesting that the eyespot develops more complex circuitry mediating aspects of photo-behavior other than phototaxis (for example, circadian entrainment).

A transgenic *Platynereis* line expressing enhanced green fluorescent protein (EGFP) under the control of the *cis*-regulatory elements of *r-opsin1* revealed the presence of pigment-associated eyelets (frontolateral eyelets) in juvenile and adult *Platynereis* (Backfisch et al. 2013). The continuous presence of *r-opsin1* in the eyespots throughout late larval stages (Fig. 2L–N), the presence of pigment, and the expression of *pax6* in the eyespot in early larval stages (Arendt et al. 2002) and in the frontolateral eyelets (Backfisch et al. 2013) argue that the frontolateral eyelets are equivalent to the eyespots.

Our phylogenetic tree suggests that the divergence of *r-opsin1* and *r-opsin3* predates the divergence of sedentary and errant annelids. We found that the two opsins are expressed in adjacent cells in the eyespots and co-express in the adult eyes. It would be interesting to test how common this is for annelids. Interestingly, the presence of two photoreceptors has been described in the eyespots of the sedentary annelids *Pectinaria koreni* and *Spirorbis spirorbis* (Bartolomaeus 1992a). Two-celled eyespots also occur in the Sipuncula (Blumer 1997), the likely sister group to the Errantia–Sedentaria clade (Struck et al. 2011). Further sampling of annelid opsins is needed to clarify the exact timing of duplication of *r-opsin1* and *r-opsin3*.

Some mollusk r-opsins form a clade (Clade II) with annelid *r-opsin1* and *r-opsin3*. Several mollusks have everse rhabdomeric eyes both in the larval and the adult stages. These eyes are morphologically similar to adult eyes of annelids (Gibson 1984). It will be interesting to test whether Clade II opsins are expressed in these molluskan eyes.

In the eyes of adult *Platynereis*, the presence of both r-opsins in the photoreceptor’s rhabdomeres, in the vicinity of the pigment cups, is consistent with their roles in detecting the direction of incoming light. Several examples of opsin co-expression are known in animals and, provided that the spectral properties are different, can broaden the spectral sensitivity of photoreceptors (Arikawa et al. 2003). The co-expression of opsins in the eyes of adult *Platynereis* may serve a similar function.

Alternatively, photoreceptors may change the opsin they express during a developmental transition, as in *Drosophila* (Sprecher and Desplan 2008). We can exclude such a developmental switch in *Platynereis*, given that the two opsins co-express in the adult eyes and are present throughout larval and adult stages.

Besides their canonical localization in the photoreceptor cell body and rhabdom, *r-opsin1* mRNA and protein are also present in the photoreceptor’s axon terminals, indicative of mRNA localization and localized translation. The localization of mRNA is a widespread and important regulatory mechanism for the post-translational regulation of gene expression in neurons and other types of cells (Martin and Ephrussi 2009). The localization of the *r-opsin1* mRNA to the axons of the photoreceptors may have functional significance in the perception of light. Since the axon terminals are not shaded by pigment, the opsin molecules present there cannot have a function in directional sensitivity. The r-opsin1 in the axon terminals may play a more general regulatory role, for example in the detection of overall ambient light intensity.

Our results highlight an unexpected functional elaboration of the eyespots of *Platynereis* larvae, suggesting that the eyespots take on a function additional to phototaxis during larval development. Furthermore, molecular, behavioral, and ultrastructural work on *Platynereis* larvae will allow the functional analysis of the six larval eyes and their opsins within the context of simple photo-behaviors of larvae.

## Funding

This work was supported by a Sequencing Grant from the Max Planck Society (M.IF.A.ENTW8050 to G.J.). The research leading to these results received funding from the European Research Council under the European Union’s Seventh Framework Programme (FP7/2007-2013)/European Research Council Grant Agreement 260821.

## Supplementary data

Supplementary Data available at *ICB* online.

Supplementary Data
