# Supplementary material for: Expression Dynamics and Protein Localization of Rhabdomeric Opsins in Platynereis Larvae
Source: Integr Comp Biol. 2013 May 10;53(1):7–16. doi: 10.1093/icb/ict046 (PMC3687135; doi:10.1093/icb/ict046)
Supplement: Supplementary Data [file supp_53_1_7__index.html]

Expression dynamics and protein localization of rhabdomeric opsins in Platynereis larvae — Supplementary Data 

# Expression Dynamics and Protein Localization of Rhabdomeric Opsins in *Platynereis* Larvae

## Supplementary Data

files

**Files in this Data Supplement:**

- Supplementary figure - tif.file
- Supplementary Data - doc file
