# Supplementary figures and images for: Expression Dynamics and Protein Localization of Rhabdomeric Opsins in Platynereis Larvae
Source: Integr Comp Biol. 2013 May 10;53(1):7–16. doi: 10.1093/icb/ict046 (PMC3687135; doi:10.1093/icb/ict046)

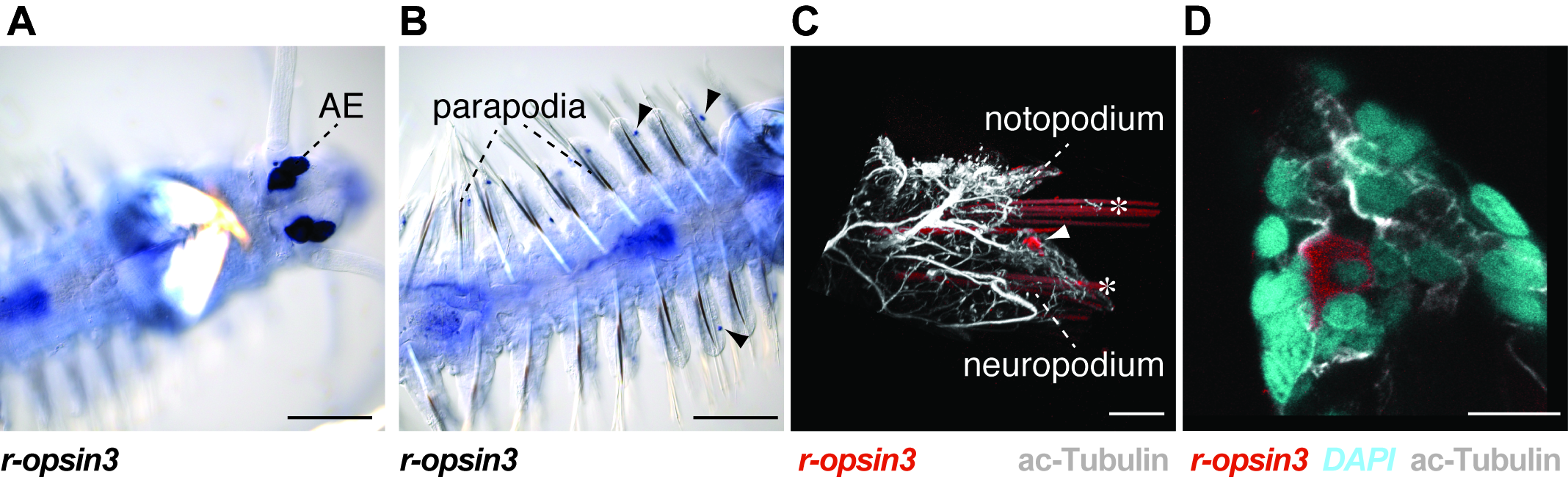

Supplement: Supplementary Data [file supp_ict046_icb-2013-0002-File007.tif]
