# Supplementary material for: Expression Dynamics and Protein Localization of Rhabdomeric Opsins in Platynereis Larvae
Source: Integr Comp Biol. 2013 May 10;53(1):7–16. doi: 10.1093/icb/ict046 (PMC3687135; doi:10.1093/icb/ict046)
Supplement: Supplementary Data [file supp_ict046_icb-2013-0002-File006.doc]

**Supplementary Figure 1**. Expression of *r-opsin3* in the adult eyes and parapodia in an adult *Platynereis*.

(A, B) Differential interference contrast (DIC) image of an adult *Platynereis* with *r-opsin3* expression in the adult eyes (A) and in the parapodia in the trunk (B). (C) *In situ* hybridization for *r-opsin3* (red) counterstained for acetylated tubulin (white) showing expression in a notopodium of an adult. (D) Close up image of the notopodium showing *r-opsin3* expression in a single cell (red), counterstained for acetylated tubulin (white) and DAPI (cyan). Asterisks in (C) show the chaetae, arrowheads in (B, C) indicate the *r-opsin3*-expressing cells in the notopodia. Scale bars: (A-D) 50 µm.
